# Editorial: Tubulinopathies: fundamental and clinical challenges

**DOI:** 10.3389/fncel.2023.1296958

**Published:** 2023-10-13

**Authors:** Antonella Sferra, Enrico Bertini, Georg Haase

**Affiliations:** ^1^Unit of Neuromuscular Disorders, Translational Pediatrics and Clinical Genetics, Bambino Gesù Children's Hospital, IRCCS, Rome, Italy; ^2^MPATHY Laboratory, Institute of Systems Neuroscience, U1106 INSERM & Aix-Marseille University, Marseille, France

**Keywords:** tubulinopathies, tubulin (microtubules), neurodegeneration, neurodevelopment, *TUBA1A*, *TUBB3*, KIF21A

Tubulinopathies are a wide family of neurological disorders caused by mutations in genes encoding tubulins, the structural units of microtubules. They represent a growing class of pathologies for which no therapies are currently available.

More than 255 tubulin mutations have been reported so far (Attard et al., 2022) and the number of tubulin mutations as well as the phenotypic spectrum of tubulinopathies are ever increasing. Our knowledge about the pathogenesis of tubulinopathies remains however fragmentary and the development of therapeutic interventions will be a huge challenge.

Our understanding about the pathogenesis of tubulinopathies is further hampered by our limited knowledge on tubulin and microtubule regulatory mechanisms at the transcriptional, translational and post-translational level (Figures 1A, B). Many of these mechanisms have recently emerged e.g., the modulation of microtubule polymerization by glutathionylation (Chen et al., 2021) but their biological significance is not yet full understood thus making it difficult to faithfully predict the effects of tubulin mutations on microtubule function.

**Figure 1:**
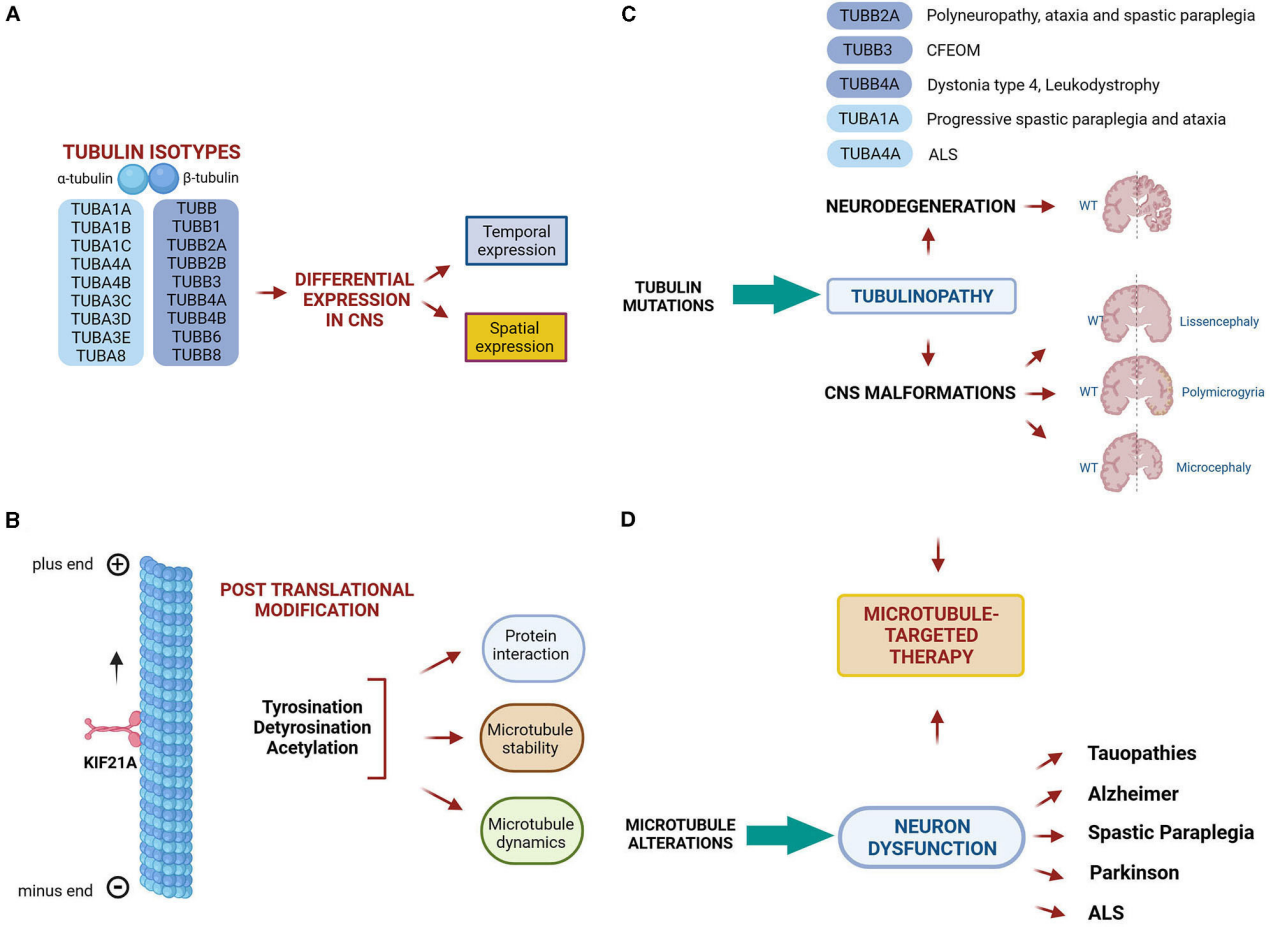
Microtubule comlexity and microtubule related diseases. **(A, B)** Microtubule complexity is generated by the expression of different α and β-tubulin isotypes and by the addition of post translational modifications, which have the potential to tune microtubule dynamics, stability and interactions with MAPS. **(C)** Mutations in tubulin genes cause a class of neurological diseases known as tubulinopathies, which include both malformations of the central nervous system and neurodegenerative disorders. **(D)** Microtubule alteration are also considered a pathomechanism underlying neuron dysfunction in a wide range of neurodegenerative diseases. **(C, D)** Thus, microtubule- based therapies represent a promising strategy to prevent or rescue microtubule dysfunction occurring in neurological disorders. The figure has been created in “BioRender.com”.

The Research Topic entitled “*Tubulinopathies: fundamental and clinical challenges*” aims to improve our knowledge about tubulinopathies, and more generally about mechanisms governing microtubule in normal and pathological conditions. The contributions of the Research Topic fall principally into two lines of research.

The first line concerns the clinical and molecular characteristics of tubulinopathies and the pathogenic effects of tubulin mutations. Tubulinopathies classically represent a broad spectrum of neurodevelopmental disorders leading to cortical malformations (Bahi-Buisson et al., 2014) (Figure 1C). Recent discoveries have demonstrated that mutations in several tubulin isotypes can cause various malformations beyond the cerebral cortex (Cederquist et al., 2012; Madrigal et al., 2019) and even trigger neurodegeneration (Smith et al., 2014; Sferra et al., 2018).

Pathogenic mutations in *TUBB3* for instance were shown to be associated with isolated or syndromic congenital fibrosis of the extraocular muscles (CFEOM) and with malformations of cortical development (MCDs) (Poirier et al., 2010; Tischfield et al., 2010). In the present Research Topic, Puri et al. provide a comprehensive review of CFEOM- and MCD-related *TUBB3* mutations, their associated phenotypes and pathomechanisms. In particular, they report that *TUBB3* missense mutations can cause errors in the growth and guidance of cranial and central axons, as well as in cortical neuronal migration and organization (Figure 1C). The effects of some *TUBB3* mutations are mimicked by specific mutations in the neuronal kinesin KIF21A, pointing to common pathogenic mechanisms (Figure 1C).

By contrast, the α-tubulin *TUBA1A* has been mainly associated with lissencephaly and a total of 121 *TUBA1A* mutations have been identified in patients with neurodevelopmental disorders (Hoff et al., 2022). Zocchi et al. describe the first *TUBA1A* mutation associated with a neurodegenerative condition characterized by ataxia and progressive spastic paraplegia. They demonstrate that the disease-causing mutation decreases TUBA1A protein stability, proposing a novel disease mechanisms in which the haploinsufficiency of this may be sufficient to drive neurodegeneration (Figure 1C). Earlier studies have shown that *TUBA4A* mutations can be responsible for rare forms of the neurodegenerative disorder amyotrophic lateral sclerosis (Smith et al., 2014).

The second line of research addresses the structural dynamics of tubulins, microtubules and microtubule-associated proteins (MAPs) as well as post translational modifications (PTMs) of tubulins in normal and pathological conditions (Figure 1B).

Hoff et al. highlight how deepening our knowledge on the interaction between tubulins, microtubules and MAPs can better predict the effects of tubulin mutations. The authors offer a description of tubulin structural dynamics and MAPs, and explain how microtubules and MAPs regulate critical steps of neurodevelopment. They further discuss how tubulin mutations can cause tubulinopathies by altering interactions between microtubules and their regulators or by impairing microtubule dynamics.

By modulating the expression of tubulin tyrosine ligase (TTL), the enzyme that catalyzes the post-translational addition of tyrosine to the detyrosinated end of α-tubulin,  Müller, Ringer et al. demonstrate that the balance of tyrosinated and detyrosinated microtubules finely regulates cell shape and adhesion in both D2 epithelial cell cultures and D3 intestinal organoids. The authors also propose that TTL has a central role in regulating the assembly of focal adhesion since its depletion prolonged the persistence of vinculin at these sites and its binding efficiency to different focal adhesion adapters.

In a companion paper, the same research group demonstrates that tyrosinated and detyrosinated microtubules exert opposite effects during cell migration of MDCK cells and MDCK cyst formation. The authors highlight a specific role of detyrosinated tubulin in promoting cell motility and direction and propose EB1, a MAP that tracks microtubule plus ends, as a key mediator during cell migration, connecting detyrosinated microtubule to cell membrane, at adhesion plaques (Müller, Gorek et al.). Dysregulation of tubulin PTMs occur in a wide range of brain disorders (Dompierre et al., 2007; Cartelli et al., 2013; Zhang et al., 2015) and both tubulin PTMs as well as some of their catalyzing enzyme have emerged as promising therapeutic targets for the treatment of neurological conditions (Rogowski et al., 2021) (Figure 1D).

Wali et al. demonstrate that peripheral blood mononuclear cells (PBMCs) of patients affected by Spastin (SPAST)-associated hereditary spastic paraplegia (HSP), exhibit reduced levels of acetylated α-tubulin, thus reflecting the pathological reduction of acetylated α-tubulin previously observed in patient-derived neuronal cells (Wali et al., 2020). They further demonstrate that the tubulin binding agent noscapine increases the level of acetylated tubulin both in patient PBMCs after *in vitro* treatment and in mouse brain following *in vivo* administration. The authors thus propose that PBMCs of SPAST-related HSP patients may represent a suitable *in vitro* model to study the pathology and the measurement of acetylated tubulin a tool to quantify the treatment effects of noscapine. A schematic representation of microtubule complexity and microtubule related diseases is shown in Figure 1.

We hope that this Research Topic will help to better understand the complex network of microtubule functions and regulations, to understand how the latter act at the cellular and sub-cellular levels, and how they are differentially affected by tubulin mutations.

In addition, we hope that the Research Topic will foster further functional and genetic studies on microtubule function and dysfunction in neurons to expand the mutational and phenotypic spectrum of tubulinopathies. These efforts will be key to design innovative microtubule-based interventions for the treatment of tubulinopathies.

## Author contributions

AS: Writing—original draft. EB: Writing—review and editing. GH: Writing—review and editing.
